# Prior SARS-CoV-2 infection rescues B and T cell responses to variants after first vaccine dose

**DOI:** 10.1126/science.abh1282

**Published:** 2021-04-30

**Authors:** Catherine J. Reynolds, Corinna Pade, Joseph M. Gibbons, David K. Butler, Ashley D. Otter, Katia Menacho, Marianna Fontana, Angelique Smit, Jane E. Sackville-West, Teresa Cutino-Moguel, Mala K. Maini, Benjamin Chain, Mahdad Noursadeghi, UK COVIDsortium Immune Correlates Network, Tim Brooks, Amanda Semper, Charlotte Manisty, Thomas A. Treibel, James C. Moon, UK COVIDsortium Investigators, Ana M. Valdes, Áine McKnight, Daniel M. Altmann, Rosemary Boyton

**Affiliations:** 1 Department of Infectious Disease, Imperial College London, London, UK.; 2 Blizard Institute, Barts and the London School of Medicine and Dentistry, Queen Mary University of London, London, UK.; 3 National Infection Service, Public Health England, Porton Down, UK.; 4 St Bartholomew’s Hospital, Barts Health NHS Trust, London, UK.; 5 Royal Free London NHS Foundation Trust, London, UK.; 6 Division of Medicine, University College London, London, UK.; 7 James Wigg Practice, Kentish Town, London, UK.; 8 Division of Infection and Immunity, University College London, London, UK.; 9 Institute of Cardiovascular Science, University College London, London, UK.; 10 Academic Rheumatology, Clinical Sciences, Nottingham City Hospital, Nottingham, UK.; 11 NIHR Nottingham Biomedical Research Centre, Nottingham University Hospitals NHS Trust and University of Nottingham, Nottingham, UK.; 12 Department of Immunology and Inflammation, Imperial College London, London, UK.; 13 Lung Division, Royal Brompton and Harefield Hospitals, London, UK.

## Abstract

Severe acute respiratory syndrome coronavirus 2 (SARS-CoV-2) vaccine rollout has coincided with the spread of variants of concern. We investigated whether single-dose vaccination, with or without prior infection, confers cross-protective immunity to variants. We analyzed T and B cell responses after first-dose vaccination with the Pfizer/BioNTech messenger RNA vaccine BNT162b2 in health care workers (HCW) followed longitudinally, with or without prior Wuhan-Hu-1 SARS-CoV-2 infection. After one dose, individuals with prior infection showed enhanced T cell immunity, antibody-secreting memory B cell response to the spike protein, and neutralizing antibodies effective against variants B.1.1.7 and B.1.351. By comparison, HCW receiving one vaccine dose without prior infection showed reduced immunity against variants. B.1.1.7 and B.1.351 spike mutations resulted in increased, abrogated, or unchanged T cell responses, depending on human leukocyte antigen (HLA) polymorphisms. Single-dose vaccination with BNT162b2 in the context of prior infection with a heterologous variant substantially enhances neutralizing antibody responses against variants.

## A boost from infection

During clinical trials of severe acute respiratory syndrome coronavirus 2 vaccines, no one who had survived infection with the virus was tested. A year after the pandemic was declared, vaccination of previously infected persons is a reality. Reynolds *et al.* address the knowledge gap in a cohort of UK health care workers given the Pfizer/BioNTech vaccine in which half of the participants had experienced natural virus infections early in the pandemic (see the Perspective by Crotty). Genotyping indicated that a genetic component underlies heterogeneity in immune responses to vaccine and to natural infection. After vaccination, naïve individuals developed antibody responses similar to those seen in naturally infected persons, but T cell responses were more limited and sometimes absent. However, antibody and memory responses in individuals vaccinated after infection were substantially boosted to the extent that a single vaccine dose is likely to protect against the more aggressive B.1.1.7 variant. It is possible that the messenger RNA vaccine has an adjuvant effect, biasing responses toward antibody generation.

*Science*, abh1282, this issue p. 1418; see also abj2258, p. 1392

## Teaser

Previous infection results in enhanced variant cross-protective T and B cell responses to a single BNT162b2 vaccine dose.

During worldwide rollout of severe acute respiratory syndrome coronavirus 2 (SARS-CoV-2) vaccines, it is vital to understand how vaccination influences immune responses and protection among those who have had prior natural SARS-CoV-2 infection. This is a knowledge gap because a history of previous infection was an exclusion criterion in phase 3 vaccine trials (*1*). Countries have adopted diverse approaches—among them, the UK policy to maximize deployment of first doses to the largest possible number of people by extending the time interval to second dose. At the end of 2020, it became apparent that several virus variants had emerged (*2*, *3*) and that these might affect vaccine rollout. The B.1.1.7 variant, possessing the spike Asn^501^→Tyr (N501Y) mutation, first emerged in the UK in December 2020 and spread rapidly (*4*). Additional variants of concern (VOC) include the B.1.351 variant, which emerged at about the same time in South Africa, and the P.1 variant, which emerged in January 2021 in Brazil. In addition to the N501Y mutation, both of these variants have the E484K mutation, which is implicated in escape from neutralizing antibodies (nAbs) (*5*, *6*).

The Pfizer/BioNTech mRNA vaccine BNT162b2 encodes a prefusion-stabilized, membrane-anchored SARS-CoV-2 full-length spike protein modified by two proline substitutions (*1*, *7*, *8*). A two-dose regimen of 30 μg BNT162b2, 21 days apart, confers 95% protection against Wuhan-Hu-1 SARS-CoV-2 (*1*), eliciting high nAb titers as well as CD4 and CD8 cell responses (*8*). When given as a single 60-μg dose, BNT162b1 induced virus Ab neutralization, but T cell responses were reduced compared with the standard prime-boost regime (*8*). A single 30-μg dose of BNT162b1 was not reported beyond day 21. However, the cumulative incidence of COVID-19 cases among 21,676 placebo and 21,699 vaccine recipients diverged 12 days after the first dose, indicating possible early-onset first-dose protection (*1*). For those who were previously infected, single-dose vaccination may act as a boost after natural infection. Therefore, we aimed to test the impact of prior SARS-CoV-2 infection on T and B cell responses to first-dose vaccination.

To do this, we analyzed T and B cell immunity after the first 30-μg dose of the Pfizer/BioNTech mRNA vaccine BNT162b2 in a cohort of UK hospital health care workers (HCW) (*9*–*12*)*.* The COVIDsortium HCW cohort has been studied longitudinally since the end of March 2020, providing accurate infection and immune history in the context of genotyping, including human leukocyte antigen (HLA) imputation (*10*–*12*). Our aim was to compare T and B cell immunity after a first dose of vaccine in December 2020 in postinfection (after natural infection), vaccinated postinfection (vaccination in the context of prior SARS-CoV-2 infection), and vaccinated naïve (single-dose vaccination) individuals. We sought to explore whether there is evidence for altered T cell recognition of the B.1.1.7 and B.1.351 variants and, in particular, of the N501Y mutation shared by several VOC.

The UK has deployed a heterodox vaccination regimen to maximize immune protection and slow spread of the B.1.1.7 lineage, giving an initial 30-μg dose of BNT162b2 followed by boosting up to 12 weeks later (*13*). A cross-sectional substudy (*n* = 51 individuals) of the existing longitudinal HCW cohort (*9*–*12*) was recruited 22 (±2) days after the first dose. After the start of the study, the majority of acute infections had already occurred among this cohort (*11*). At the time of receiving their first vaccine dose in December 2020, prior to the emergence of VOC, 25 individuals were ~39 weeks removed from SARS-CoV-2 infection with the Wuhan-Hu-1 strain, and 26 were confirmed uninfected, having tested negative in longitudinal serology for spike and nucleocapsid (N) proteins (table S1 and fig. S1).

**Fig. 1:**
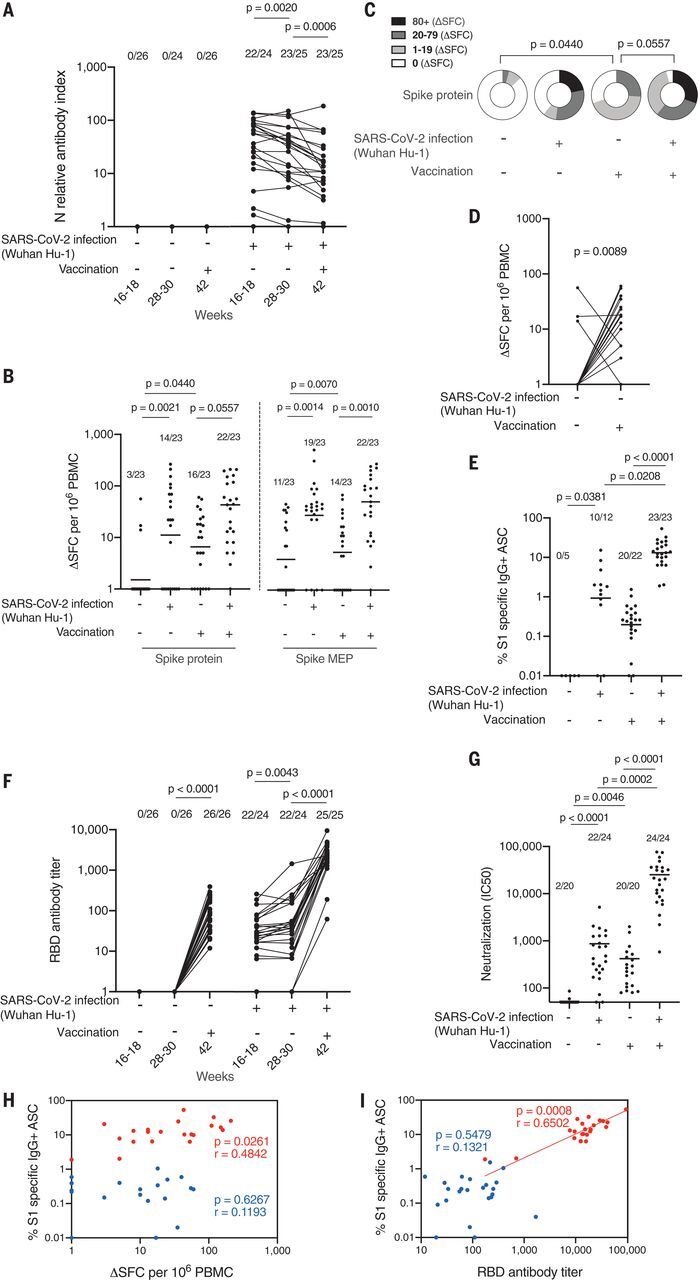
Impact of prior natural infection with SARS-CoV-2 during the first wave on T and B cell responses to a single dose of the mRNA SARS-CoV-2 vaccine BNT162b2. (**A**) Nucleocapsid Abs measured by electrochemiluminescence immunoassay analyzer (ECLIA) in serum samples from HCW with (*n* = 25 individuals) and without (*n* = 26 individuals) laboratory-confirmed SARS-CoV-2 infection (Wuhan-Hu-1, during the first wave) 3 weeks after a single dose of the mRNA SARS-CoV-2 vaccine BNT162b2. (**B**) Magnitude of T cell response to spike protein and spike mapped epitope peptides (MEPs) in HCW with and without laboratory-confirmed SARS-CoV-2 infection (*n* = 23 per group). Data are shown prevaccination (16 to 18 weeks after infection) and 3 weeks after the first-dose vaccination (week 42) with line at geometric mean. (**C**) Proportion of HCW with (*n* = 23) and without (*n* = 23) laboratory-confirmed SARS-CoV-2 infection (during the first wave) with a T cell response to spike protein within the range of 0, 1 to 19, 20 to 79, and >80 ΔSFC/10^6^ PBMC before and 3 weeks after first-dose vaccination. (**D**) Magnitude of T cell response to spike protein in HCW without a history of SARS-CoV-2 infection, plotted pairwise at 16 to 18 weeks and 42 weeks (3 weeks after first-dose vaccination). (**E**) Percentage of S1-specific IgG^+^ antibody-secreting cells (ASCs) in vaccinated HCW with (*n* = 23) and without (*n* = 22) prior SARS-CoV-2 infection and in unvaccinated HCW with (*n* = 12) and without (*n* = 5) prior infection. Line at geometric mean. (**F**) RBD Ab titers measured by ECLIA in serum samples from HCW with (*n* = 25) and without (*n* = 26) laboratory-confirmed SARS-CoV-2 infection following first-dose vaccination. (**G**) Neutralizing antibody titer (IC_50_) against Wuhan-Hu-1 authentic virus in HCW with (*n* = 24) and without (*n* = 20) laboratory-confirmed SARS-CoV-2 infection. Line at arithmetic mean. (**H**) Correlation between percentage of S1-specific ASC and magnitude of T cell response to spike protein in vaccinated HCW with (*n* = 21, red) and without (*n* = 19, blue) a history of SARS-CoV-2 infection during the first wave. (**I**) Correlation between percentage of S1-specific ASC and RBD Ab titer in HCW with (*n* = 23, red) and without (*n* = 23, blue) a history of SARS-CoV-2 infection. [(A), (B), (E), and (F)] Numbers of HCW in each group with detectable responses are shown. [(F) and (G)] Data are shown prevaccination (16 to 18 weeks after infection) and 3 weeks after the first-dose vaccination (week 42). [(A), (D), and (F)] Wilcoxon matched-pairs signed rank test. [(B), (C), (E), and (G)] Kruskal Wallis multiple comparison analysis of variance (ANOVA) with Dunn’s correction. [(H) and (I)] Spearman’s rank correlation. Ab, antibody; HCW, health care workers; RBD, receptor binding domain; S1, spike subunit 1; SFC, spot forming cells.

We first measured SARS-CoV-2 N antibody longitudinally up to 16 to 18 weeks, then at 28 to 30 weeks, and finally at 42 weeks after recruitment, to confirm that there was no laboratory evidence of new infection at the time of drawing blood for the vaccine study at 42 weeks; none of the previously uninfected HCW had become seropositive (Fig. 1A). T cell responses to spike protein and mapped epitope peptides (MEPs) in either postinfection, vaccinated postinfection, and vaccinated naïve individuals were compared (Fig. 1B). Ninety-six percent (22/23) of vaccinated postinfection individuals mounted a T cell response to spike protein compared with 70% (16/23) of vaccinated naïve individuals, with a fourfold increase in the magnitude of the T cell response. Furthermore, while the T cell response to spike protein in vaccinated naïve individuals increased (*P* = 0.0440), it was lower than that of vaccinated postinfection individuals (*P* = 0.0557) (Fig. 1C). As expected, there was no significant change in T cell response to N (a measure of immunity to natural infection) (fig. S2A).

Paired analysis of T cell immunity to spike protein in previously uninfected individuals, analyzed at the 16- to 18-week time point and 3 weeks after vaccination, showed a significantly increased response (*P* = 0.0089) (Fig. 1D). Three individuals who previously showed a response, despite lack of laboratory evidence for infection (therefore presumably a cross-reactive response to an endemic human coronavirus), showed an unchanged or decreased response to spike after vaccination.

The size of the SARS-CoV-2 spike subunit 1 (S1)–specific memory B cell (MBC) pool was investigated by B cell enzyme-linked immunosorbent spot (ELISpot) assay (Fig. 1E and fig. S2B). As for T cell responses, the number of S1-specific immunoglobulin G (IgG^+^) antibody-secreting cells (ASCs) was far greater in vaccinated postinfection individuals than in vaccinated naïve individuals (*P* < 0.0001). Prior infection generated a 63-fold increase in S1-specific ASCs. There were no preexisting S1-specific ASCs in uninfected HCW before vaccination. Twenty of 22 vaccinated naïve individuals had detectable S1-specific ASCs composing 0.02 to 1.54% of the MBC pool. By comparison, all vaccinated postinfection individuals had detectable S1-specific ASCs (1.90 to 50% of the MBC pool). We previously reported (*14*) spike receptor binding domain (RBD) enhanced Ab responses in the vaccinated postinfection group. In this work, the vaccinated naïve group attained antibody titers similar to those of the postinfection group at 16 to 18 weeks and 28 to 30 weeks (Fig. 1F). Vaccinated naïve individuals demonstrated a lower nAb response to wild-type virus than was seen after natural infection at 16 to 18 weeks, although this did not achieve statistical significance. In line with the findings for MBC and RBD binding, there was a significantly enhanced nAb response in vaccinated postinfection individuals compared with the vaccinated naïve group (Fig. 1G), with a mean value of 25,273 compared with 420, that is, a 60-fold increase. To put this in context, these values are 43-fold higher than the values recorded after two vaccine doses in the phase 1 trial (*7*). There was no correlation between the magnitude of the spike protein T cell response and the percentage of S1-specific ASCs (Fig. 1H). As expected, there was a positive correlation between the percentage of S1-specific ASCs and the serum titer of RBD antibody in the vaccinated postinfection individuals [correlation coefficient (*r*) = 0.6502; *P* = 0.0008] (Fig. 1I). After vaccination, two previously infected individuals showed lower percentages of S1-specific memory B cells and reduced serum RBD-specific antibody levels than the rest of the group; prior infection involving case-definition symptoms tended to be associated with a higher specific B cell frequency than milder disease (Fig. 1F and fig. S2C). These individuals who, despite infection, had also not shown a detectable T cell response (one never seroconverted, and the other rapidly became seronegative during longitudinal follow-up) had a poor or absent response to infection that was only minimally overcome by vaccination.

The data in Fig. 1 indicate that there is a strong prime-boosting effect of prior infection on single-dose vaccination. Augmentation is seen more strongly in MBC frequency, anti-RBD, and nAb responses than for T cell response frequency. Furthermore, there was no correlation between S1 ASC frequency and T cell response frequency (Fig. 1H). There is, however, a correlation between S1 ASC and RBD antibody titers, indicating that individuals with higher numbers of MBCs mount stronger antibody responses, and individuals who had experienced infection clustered at the higher end of this response (Fig. 1I).

**Fig. 2:**
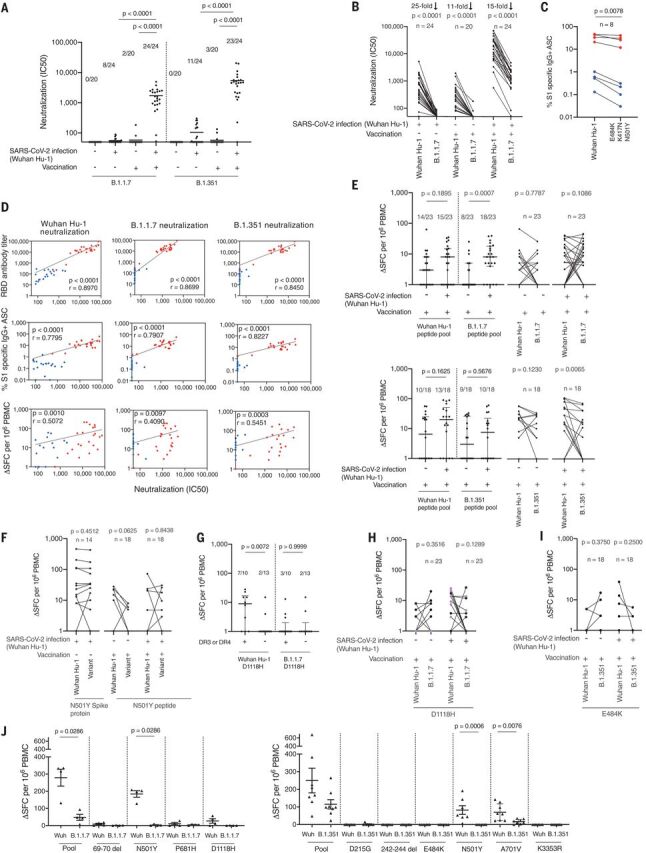
Impact of vaccination and prior natural infection with SARS-CoV-2 during the first wave on T and B cell responses to the UK B.1.1.7 and South African B.1.351 variants. (**A**) Neutralizing antibody (nAb) titer (IC_50_) against B.1.1.7 and B.1.351 authentic virus in HCW with (*n* = 24) and without (*n* = 20) laboratory-confirmed SARS-CoV-2 infection (Wuhan-Hu-1). Lines at arithmetic mean. Data are shown prevaccination (16 to 18 weeks after infection) and 3 weeks after the first-dose vaccination (week 42). (**B**) nAb (IC_50_) titers against Wuhan-Hu-1 and B.1.1.7 authentic viruses plotted pairwise by individual. (**C**) Percentage of Wuhan-Hu-1 S1 and S1 containing variant mutations (E484K, K417N, and N501Y) specific IgG^+^ antibody-secreting cells (ASCs) in vaccinated HCW with (*n* = 4) and without (*n* = 4) prior SARS-CoV-2 infection. Single-letter abbreviations for the amino acid residues are as follows: A, Ala; C, Cys; D, Asp; E, Glu; F, Phe; G, Gly; H, His; I, Ile; K, Lys; L, Leu; M, Met; N, Asn; P, Pro; Q, Gln; R, Arg; S, Ser; T, Thr; V, Val; W, Trp; and Y, Tyr. (**D**) Correlations between nAb (IC_50_) titers of Wuhan-Hu-1, B.1.1.7, or B.1.351 authentic virus and RBD Ab titer, percentage of S1-specific ASC, and magnitude of T cell response to S1 protein in vaccinated HCW with (*n* = 22 to 24, red) and without (*n* = 18 to 20, blue) a history of SARS-CoV-2 infection. (**E**) Magnitude of T cell response to Wuhan-Hu-1, B.1.1.7, or B.1.351 peptide pools in vaccinated HCW with (*n* = 23 or 18) and without (*n* = 23 or 18) SARS-CoV-2 infection (Wuhan-Hu-1), plotted as grouped data (median plus interquartile range) and pairwise for each individual. (**F**) Magnitude of T cell response to Wuhan-Hu-1 S1 protein and N501Y variant spike RBD protein in unvaccinated HCW with laboratory-confirmed SARS-CoV-2 infection (*n* = 14) or to Wuhan-Hu-1 and N501Y mutated peptide in vaccinated HCW with (*n* = 18) and without (*n* = 18) a history of SARS-CoV-2 infection, plotted pairwise by individual. (**G**) Magnitude of T cell response to Wuhan-Hu-1 or B.1.1.7 D1118H peptide in vaccinated HCW with a history of SARS-CoV-2 infection (*n* = 23), plotted by DRB1*0301 or DRB1*0401 status. Lines at median plus interquartile range. (**H**) Magnitude of T cell response to Wuhan-Hu-1 or B.1.1.7 D1118H peptide in vaccinated HCW with (*n* = 23) and without (*n* = 23) a history of SARS-CoV-2 infection, plotted pairwise by individual and with individuals carrying DRB1*0301 or DRB1*0401 alleles marked in purple. (**I**) Magnitude of T cell response to Wuhan-Hu-1 or B.1.351 E484K mutated peptide in vaccinated HCW with (*n* = 18) and without (*n* = 18) a history of SARS-CoV-2 infection, plotted pairwise by individual. (**J**) Magnitude of T cell response to Wuhan-Hu-1 (Wuh), B.1.1.7, or B.1.351 peptide pools and individual peptides in Wuhan-Hu-1 peptide immunized HLA-DRB1*04:01 transgenic mice (left-hand panel, *n* = 4; right-hand panel, *n* = 8; lines at arithmetic mean + SEM). (A) Kruskal Wallis multiple comparison ANOVA with Dunn’s correction. [(B), (C), (E) (right-hand panels), (F), (H), and (I)] Wilcoxon matched-pairs signed rank test. (D) Spearman’s rank correlation. [(E) (left-hand panels), (G), and (J)] Mann-Whitney *U* test. ASC, antibody-secreting cells; HCW, health care workers; RBD, receptor binding domain; S1, spike subunit 1; SFC, spot forming cells.

Shortly before the vaccination program was initiated, several VOC emerged, including B.1.1.7. This variant has nine mutations in the spike protein. Several studies have reported weaker nAb responses to B.1.1.7 relative to the previously circulating Wuhan-Hu-1 strain (*2*–*6*, *15*–*18*). The majority of SARS-CoV-2 immune naïve individuals made no nAb response to the B.1.1.7 (18/20) and B.1.351 (17/20) variants after single-dose vaccination. In contrast, almost all vaccinated postinfection individuals made a strong nAb response to the B.1.1.7 (24/24) and B.1.351 (23/24) variants after a single-dose vaccination, with a 46-fold (B.1.1.7) and 63-fold (B.1.351) increase in mean nAb half-maximal inhibitory concentration (IC_50_) in vaccinated postinfection individuals compared with vaccinated naïve individuals. In a paired analysis, we observed in vitro significantly reduced nAb potency to authentic B.1.1.7 variant (mean: 35) with a 96% fall compared to that of Wuhan-Hu-1(mean: 866; *P* < 0.0001) in sera from individuals with a past medical history of natural infection (Fig. 2B). Worryingly, after single-dose vaccination, 90% (18/20) of vaccinated naïve individuals showed no detectable nAbs (IC_50_ < 50) against B.1.1.7 (mean IC_50_: 37; range: 0 to 184; *P* = 0.2090), but they did show demonstrable nAb responses to Wuhan-Hu-1 SARS-CoV-2 virus (mean IC_50_: 420; range: 80 to 2004; *P* = 0.0046). In contrast, all vaccinated postinfection individuals responded to single-dose vaccination with substantially enhanced nAb responses, neutralizing not just Wuhan-Hu-1 SARS-CoV-2 (mean IC_50_: 25,273; range: 581 to 76,369) but also the B.1.1.7 (mean IC_50_: 1717; range: 52 to 4919) and B.1.351 (mean IC_50_: 5451; range: 41 to 20,411) variants (Fig. 2, A and B, and fig. S3). We show a 93% reduction in neutralization (IC_50_) responses to the SARS-CoV-2 B.1.1.7 variant (mean: 1717) compared with the Wuhan-Hu-1 (mean: 25,273) virus in vaccinated postinfection individuals. However, despite this fall, the majority (22/24) remain within a “protective threshold.” This was not the case for vaccinated naïve individuals. There was a 91% reduction in neutralization (IC_50_) responses against the SARS-CoV-2 B.1.1.7 variant (mean: 37) compared with the Wuhan-Hu-1 virus (mean: 420), resulting in the majority of individuals (19/20) falling below the “protective threshold.” This result was mirrored in the SARS-CoV-2 S1-specific MBC pool, where reduced numbers of S1-specific IgG^+^ ASC are seen (in vaccinated naïve individuals compared with vaccinated postinfection individuals) responding to S1 antigen containing the N501Y, K417N, and E484K mutations. Prior infection substantially enhances the specific MBC pool after single-dose vaccination (Fig. 2C). We looked at correlations between RBD binding antibodies, B cell responses, T cell responses, and IC_50_, comparing neutralization of Wuhan-Hu-1, B.1.1.7, and B.1.351 live virus (Fig. 2D). Despite the lower neutralization of B.1.1.7 and B.1.351 variants, the pattern was retained of strong correlation between RBD antibody titer and S1-specific B cell frequency and neutralization and somewhat weaker correlation between T cell response and neutralization.

A lack of Ab-mediated protection in single-dose vaccinees could be mitigated by a broader repertoire of T cell responses (*18*). To investigate differences in T cell recognition, we designed peptide pools covering the affected regions of Wuhan-Hu-1, B.1.1.7, and B.1.351 variant sequence (table S2). We compared T cell responses to these peptide pools in peripheral blood mononuclear cells (PBMCs) from vaccinated postinfection and vaccinated naïve individuals (Fig. 2E). Responses in postinfection vaccinees were in general higher than in the vaccinated naïve individuals (note an enhanced response to the B.1.1.7 peptide pool). T cell responses were heterogeneous; responses to variant pools could be either higher or lower than to Wuhan-Hu-1 pools. Alterations in affinity for the T cell receptor can lead to altered peptide ligand effects and differential polarization of cytokine effector programs, as we have previously observed in Zika virus infection (*19*). We wondered whether this was also occurring for SARS-CoV-2; however, we found no evidence for immune deviation to interleukin (IL)–4, IL-5, IL-10, IL-13, IL-17A, or IL-23 (fig. S3).

For B.1.1.7 and B.1.351, attention has centered on the N501Y mutation, as this is implicated in altered angiotensin-converting enzyme 2 (ACE2) binding and enhanced infectivity and transmission but is also a target for B and T cell recognition. We initially looked at T cell responses after natural infection and found that at 16 to 18 weeks postinfection, the N501Y mutation appeared to have no substantial differential impact on the T cell response (Fig. 2F), unlike nAb recognition (*5*).

The specific impact of any T cell epitope changes on the immune response against VOC depends on changes in peptide binding to the peptide-presenting HLA molecules. Because the HLA complex is the most polymorphic part of the human genome, any alteration to core HLA binding motifs will differentially affect people with certain HLA alleles over others. We performed in silico analysis (using NetMHCIIpan) to predict which of the B.1.1.7 and B.1.351 mutations were found in HLA core binding motifs and how this might affect binding to common HLAII alleles (DRB1*0101, DRB1*0301, DRB1*0401, DRB1*0701, DRB1*1101, DRB1*1301, and DRB1*1501) (tables S3 and S4). Some of the mutations did not fall in a region predicted to bind the HLAII alleles tested (D3L, T716I, T1001I, A1708D, and 3675-7 SGF del). Although several mutations were not predicted to significantly change affinity for the HLAII alleles, others did show predicted differential affinities depending on host HLAII type (tables S3 and S4). Analyzing altered responses to the D1118H mutation, we noted that individuals who carried DRB1*0301 and DRB1*0401 showed enhanced T cell responses to the Wuhan-Hu-1 peptide compared with those who did not (*P* = 0.0072) (Fig. 2G). T cell responses to the variant peptide appeared to be reduced in individuals carrying DRB1*0301 and DRB1*0401 (Fig. 2H). There is a basis for this in terms of differential HLAII binding as the D-to-H mutation is predicted to lose the T cell epitope for people carrying DRB1*0301 and DRB1*0401 but not, for example, in those who carry DRB1*0701 or DRB1*1501, who would be predicted to show an enhanced response (table S3). People carrying DRB1*1301 are predicted to gain a response as a consequence of this mutation. Analyzing responses to the E484K mutation seen in B.1.351 and P.1 variants, we noted that it did not fall in a region predicted to bind the HLAII alleles tested (table S4). The mutation appeared to have no substantial or differential impact on T cell responses (Fig. 2I).

When we primed transgenic mice expressing human HLA-DRB1*0401 with the Wuhan-Hu-1 peptide pool, T cell responses to the B.1.1.7 variant peptide pool were significantly reduced (*P* = 0.0286) (Fig. 2J). Furthermore, the T cell response to the spike N501Y mutation common to all three of the current VOC was ablated.

In this HCW cohort, vaccinated naïve individuals made an anti-S1 RBD Ab response with a mean titer of ~100 U/ml at 22 (±2) days after vaccination, roughly equivalent to the mean peak Ab response after natural infection (*14*). However, the spike T cell response after one dose was lower than after natural infection, and for 30% of vaccinees, no response could be measured. However, T cell responses are enhanced fourfold in those vaccinated postinfection. This T cell enhancement is small relative to the 63-fold change in ASCs and the corresponding 140-fold change in Roche anti-S (RBD) Ab levels we observed after one vaccine dose in HCW vaccinated postinfection (*14*). While much has been written about the impact of rapidly waning serum antibodies, our findings confirm that MBCs are nevertheless primed and able to contribute a rapid, large response to repeat exposure. The rather large effect on B cell priming and restimulation, relative to T cells, in previously infected single dose–vaccinated individuals may reflect the fact that, among the nuanced differences between the licensed SARS-CoV-2 vaccines, aspects of the mRNA adjuvant effect appear to skew immunity to high nAb titers, which may underpin its high efficacy. Our evidence for enhanced vaccine responses after infection supports the case that only one vaccine dose is necessary to maximize immune protection for SARS-CoV-2–experienced individuals (*14*, *20*).

It is notable that the high IC_50_ titers in those vaccinated after infection provide such a large protective margin that responses to authentic B.1.1.7 and B.1.351 variants are also high. In contrast, nAb responses in individuals several months on from mild infection show much lower IC_50_ titers against B.1.1.7 and B.1.351, often less than 100. Similarly, the majority of responses in naïve individuals after one dose show weak recognition of B.1.1.7 and B.1.351. This finding indicates potentially poor protection against B.1.1.7 and B.1.351 in individuals who have experienced natural infection or who have only had one vaccine dose.

It is important to map the effect of VOC mutations on any evasion of T cell immunity. The case has been made that reductions in antibody neutralization of mutant spike may be mitigated by protective T cells (*8*). A case has been made for the role of T cells as correlates of protection (*21*). Our evidence from this analysis 22 (±2) days after one dose is that T cell immunity is mostly variably low but also relatively unperturbed by the N501Y mutation. The other mutations we considered that overlay CD4 epitopes were, as might be predicted, distributed across the range of HLAII polymorphisms. Those alleles associated with loss of CD4 response to the variant pool tended to be those with a lysine in pocket 4 of the groove (HLA-DR residue 71β), whereas those with an increased response to the variant pool tended to be those with a smaller amino acid, alanine. In HLA-DRB1*0401 transgenics, we confirmed that in the context of a given HLAII heterodimer, the N501Y mutation can result in ablation of this part of the T cell response, demonstrating that HLA polymorphisms are likely to be significant determinants of responder and nonresponder status with respect to vaccine escape.

SARS-CoV-2 immunity now encompasses postinfection plus either zero, one, or two vaccine doses and first and second dose naïve vaccinated. Single-dose vaccination after infection achieves similar levels of S1 RBD binding antibodies to two doses in naïve vaccinated individuals and second-dose vaccination in one-dose vaccinated postinfection individuals offers no additional enhancement (*22*). Moving forward, it will be important to resolve the quantitative and qualitative differences between these groups in terms of neutralizing antibody repertoire as well as phenotype and durability of memory B and T cell responses. Durability of immunity to natural infection and after vaccination as well as sustained vaccine efficacy and vaccine escape need to be monitored over time.

## Supplementary Materials

science.sciencemag.org/content/372/6549/1418/suppl/DC1
Materials and Methods Figs. S1 to S4 Tables S1 to S5 UK COVIDsortium Investigators Collaborator List UK COVIDsortium Immune Correlates Network Collaborator List References (*23*–*28*) MDAR Reproducibility Checklist

## Appendix

View/request a protocol for this paper from *Bio-protocol*.
